# Delta-modulated cortical alpha oscillations support new knowledge generation through memory integration

**DOI:** 10.1016/j.neuroimage.2021.118600

**Published:** 2021-09-23

**Authors:** Nicole L. Varga, Joseph R. Manns

**Affiliations:** a Department of Psychology, Emory University, Atlanta, GA 30322, USA; b Center for Learning and Memory, The University of Texas at Austin, Austin, TX 78712, USA

**Keywords:** Cross-frequency coupling, Episodic memory, Knowledge representation, Prefrontal cortex, Reactivation, Semantic memory

## Abstract

The ability to generate new knowledge depends on integration of separate information. For example, in one episode an individual may learn that apple seeds are called pips. In a separate episode, the individual may then learn that pips contain cyanide. Integration of the related facts in memory may then support derivation of the new knowledge that apple seeds contain cyanide. Past studies show that adults form relational memories that represent the commonalities among discrete events, and that such integrated representation supports the ability to infer new knowledge. Although these integrated representations are thought to result from linking separate memories to the same neuronal ensemble, the neural mechanisms that underlie formation of such linkages are not well understood. Here we examined whether self-derivation of new, integrated knowledge was supported by oscillatory coherence, a means of linking discrete neuronal ensembles. Cortical alpha coherence was greater when adults encoded new facts that could be integrated with existing knowledge, relative to encoding unrelated facts, particularly in participants who showed better performance on the subsequent test of knowledge generation via fact integration. In high performers, posterior alpha amplitude was also modulated by delta phase, a form of cross-frequency coupling previously implicated in coordinating information stored widely throughout the cortex. Examination of the timing and topography of these respective signatures suggested that these oscillatory dynamics work in concert to encode and represent new knowledge with respect to prior knowledge that is reactivated, thus revealing fundamental mechanisms through which related memories are linked into integrated knowledge structures.

## 1. Introduction

The question of how interconnected knowledge structures are formed is fundamental to our understanding of human behavior (Bauer and Varga, 2017; Preston and Eichenbaum, 2013; Schlichting and Preston, 2015). In particular, accumulation of knowledge about the world depends on storage and organization of individual facts learned across separate episodes. Integration of incoming information with prior memories also supports the ability to extend beyond experience to generate new knowledge (Bauer and Jackson, 2015; Varga and Bauer, 2017a, 2017b). Prominent theories suggest that memory integration results when new experience is encoded by the same neuronal ensembles that represent prior, related memories, increasing the likelihood that separate yet related information may be combined in new ways (Morton et al., 2017; Ritvo et al., 2019). In line with this view, research has shown that neural activity to related events becomes more similar following learning (Cai et al., 2016; Molitor et al., 2021; Schlichting et al., 2015). Moreover, such neural similarity—which is thought to reflect integration, or linking, of previously learned information —supports the ability to infer connections among related events (Collin et al., 2015). However, there is relatively little evidence informing the precise neural computations that enable linking of separate yet related memories to the same neuronal ensemble during learning.

There is a growing consensus that neuronal oscillations play a key role in linking neurons into assemblies (Buzsáki, 2006; Buzsáki and Draguhn, 2004). Neuronal oscillations reflect rhythmic fluctuations whereby a neural ensemble’s propensity for synaptic transmission rises and falls in accord with excitability peaks and inhibitory troughs. Therefore, when oscillatory waves among neurons rise and fall in a temporally synchronous fashion, spike-timing dependent plasticity between them is facilitated, thus providing a means of strengthening the connection between discrete neuronal ensembles (Bastiaansen et al., 2011; da Silva, 2013; Fries, 2005). Although previous work shows that neuronal ensembles representing prior memories are spontaneously reactivated during encoding of new, related events (Schlichting et al., 2014; Varga and Bauer, 2017a; Zeithamova et al., 2012), less is known about how these discrete traces of information are linked in memory. In light of the known role of neuronal oscillations in strengthening synaptic connections among discrete neurons, here we examined whether oscillatory patterns at the time of learning were associated with successful knowledge generation through memory integration. Notably, semantic knowledge is represented throughout the entire cortex (Barsalou, 2008; Huth et al., 2012; Martin, 2007), such that reactivation of prior memories is evidenced through widely distributed neocortical patterns (Polyn et al., 2005; Schlichting et al., 2014; Zeithamova et al., 2012). Examination of the neural computations that enable linking of related memories to the same ensemble thus requires recording of oscillations across the entire cortex simultaneously, which can be achieved through scalp-based electrophysiology (EEG).

One view is that memory integration may rely on modulation of specific oscillations presumed to correspond generally to cortical inhibition (Ritvo et al., 2019). Extending this view to memory integration, reductions in the amplitudes of these oscillations may reflect cortical disinhibition and thus reactivation of prior memories during learning of related episodes. Indeed, recent work has directly linked levels of neocortical disinhibition to co-activation of related memories during new learning (Koolschijn et al., 2019). One oscillation that was initially thought to mediate the level of cortical inhibition is the alpha (9–12 Hz) rhythm (Berger, 1929; Klimesch, 1996, 2006; Ray and Cole, 1985), an idea that originated from the early observation that alpha amplitude increased when individuals closed their eyes or disengaged from cognitive tasks (Berger, 1929). More recently, levels of cortical alpha amplitude, as measured by EEG alpha power, have been found to correlate with several cognitive processes, including memory encoding and retrieval (Hanslmayr et al., 2012). Moreover, the emerging consensus that neuronal oscillations reflect periodic network states of both inhibition and excitation would argue against associating any oscillatory frequency with only inhibition. That is, although changes in alpha power may coarsely correlate with changes in cortical excitability, and possibly retrieval of related memories during new learning, examination of mean alpha amplitudes disregards periodicity and phase information, information that would be essential to understanding the role of oscillations in forming integrated neuronal ensembles (Klimesch et al., 2010; Palva and Palva, 2007, 2011).

Based on the widely-held view that synchronized oscillations among ensembles of neurons can facilitate synaptic transmission and plasticity (Bastiaansen et al., 2011; Buzsáki and Draguhn, 2004; da Silva, 2013; Fries, 2005), there is strong justification for hypothesizing that cortical alpha synchrony might be important for integrating ensembles representing separate memories. Moreover, cortical alpha synchrony, which can be measured by channel-to-channel alpha coherence (Manns et al., 2018), has been shown to coordinate interactions between adjacent and non-adjacent cortical areas (Sadaghiani and Kleinschmidt, 2016), thus underscoring the critical role of oscillatory synchrony in mediating large-scale cortical integration (Palva and Palva, 2011). Accordingly, we hypothesized that cortical alpha synchrony might also be an important neural underpinning of memory integration, providing a sensitive index of the linking of distributed cell assemblies that represent new and remembered information during learning.

Delta (1.5–3.5 Hz) oscillations have also been implicated in cortical integration (Palva and Palva, 2011). Furthermore, recent studies in humans have indicated that the amplitude of faster alpha oscillations wax and wane across the phases of the slower delta wave (Helfrich et al., 2017; Wöstmann et al., 2016), a type of cross-frequency coupling (CFC) that has been shown to support more complex forms of perception, learning, and cognition (Canolty & Knight, 2010; Friese et al., 2013; Tort et al., 2009). Of relevance to the present work, phase-amplitude CFC has been proposed as a basis for regulating synaptic plasticity across distributed cortical areas. That is, by time-locking faster, spike-based computations carried out in local cortical regions to the phase of a slower oscillation, such hierarchical oscillatory interactions are well suited to coordinate simultaneous activation of information stored across multiple cortical areas. Because memory integration depends on encoding new information with respect to prior, semantic knowledge that is represented across distributed cortical regions (Barsalou, 2008; Huth et al., 2012; Martin, 2007), delta-alpha cross-frequency interactions may thus further support this form of integrative learning.

**Fig. 1.**
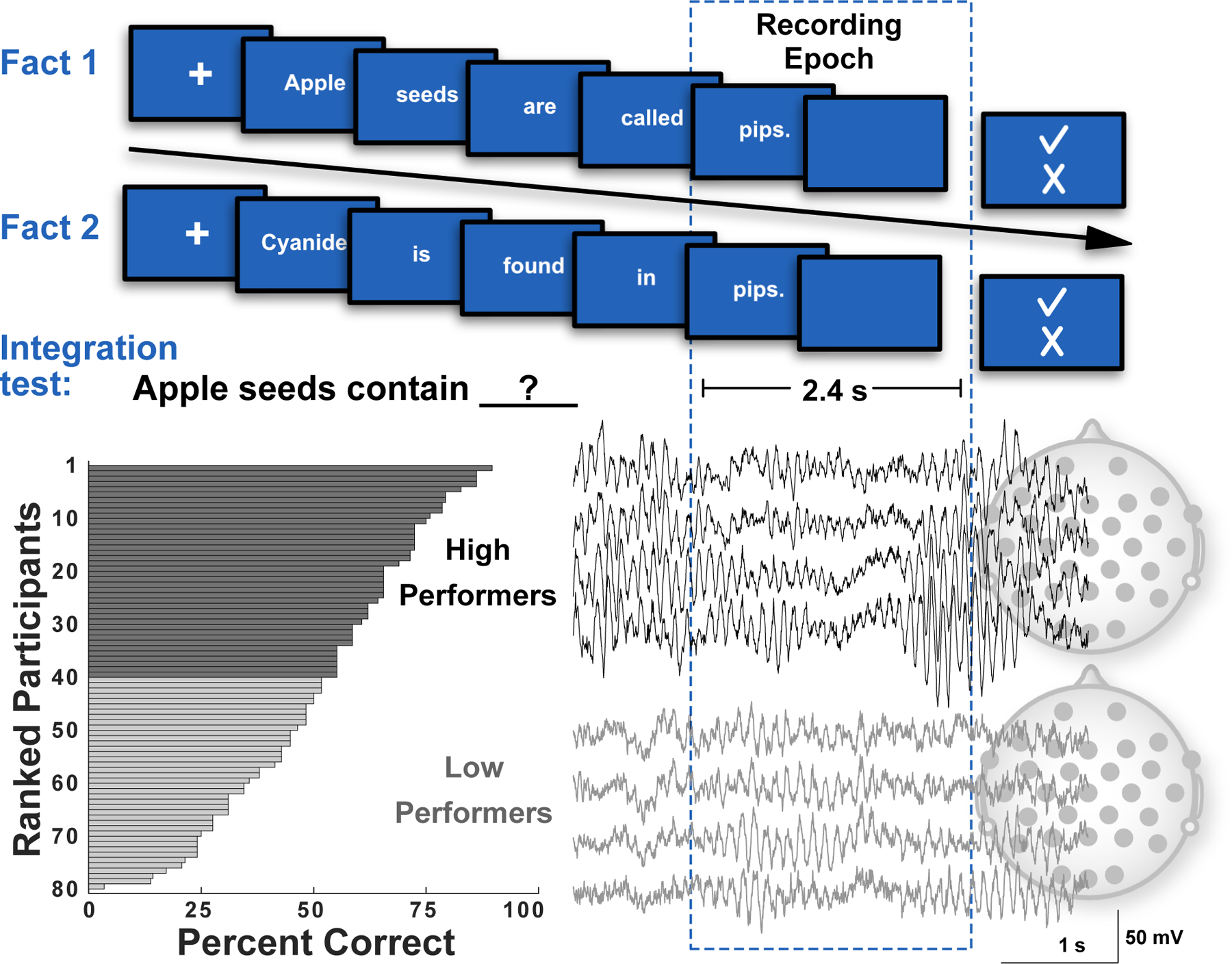
A procedure schematic shows the overall approach. The scalp EEG recording epoch started with the final word in each pair of facts. Participants were labeled as high performers or low performers based on a median split of their subsequent integration test performance, the range for which was 90% to 3% correct. Example EEG recordings from four recording channels (Fz, Cz, Pz, and Oz; out of 30) for one high performer and one low performer show that oscillations in the alpha range (9–12 Hz) were prominent. Analyses focused on alpha oscillations across all channels, split by participants’ ranked success in deriving the target integration facts through open-ended testing (high versus low performers). Following presentation of each fact, which was presented one word at a time (400 ms per word), participants were asked to indicate yes (checkmark) or no (X) as to whether they already knew the fact. A total of 30 fact pairs were presented. Related facts from a corresponding pair were separated by 2–4 intervening facts.

The present study investigated the role of both cortical alpha coherence and delta-alpha CFC in memory integration. To this end, scalp-based EEG was measured as participants read related fact pairs (e.g., *Apple seeds are called pips; Cyanide is found in pips*) that could be integrated to self-derive novel integration facts (*Apple seeds contain cyanide).* A large range of performance was observed on a subsequent test of knowledge generation through integration (Fig. 1), raising the key question of whether cortical alpha coherence and delta-alpha CFC explained such individual differences. Analyses of scalp-based EEG focused on the learning phase, particularly on the final word in each fact sentence, as those words offered links between the related pairs. Guided by previous approaches, we examined both within-subject indices of memory integration (i.e., related versus initial events from a pair) (Varga and Bauer, 2017a; Zeithamova et al., 2012), as well as between-subject differences in memory integration (i.e., high versus low performers) (Shohamy and Wagner, 2008; Zeithamova and Preston, 2010). Participants who performed well on the knowledge generation test showed greater cortical alpha coherence and delta-alpha CFC compared to participants who performed poorly. Moreover, alpha coherence was specifically increased in high performers when reading the second fact sentence in each pair, suggesting that alpha coherence played a specialized role in processing content that could be integrated with prior, reactivated knowledge. The findings are discussed with respect to their implications for understanding the oscillatory dynamics that underlie binding of separate yet related memories, and by extension, productive generation of new semantic knowledge.

## 2. Materials and methods

### 2.1. Participants

Participants were 80 adults aged 18 to 24 years (*M* = 19.81 years; *SD* = 1.22 years; 47 identified as female and 33 identified as male) recruited through introductory psychology courses at a private university. Participants completed two sessions spaced 6–8 days apart (*M delay* = 6.91 days). The current report focuses on electrophysiological and behavioral measurements collected during the first visit. An additional 28 participants were tested but excluded from analysis due to excessive noise in the electrophysiological recordings (*N* = 22; 11 for motion, 4 for muscle artifact, 4 for unacceptable impedance levels, and 3 for bridging of conductive gel between channels), a history of neurological impairment (*N* = 3), experimenter error (*N* = 2), and diagnosis of a learning disability which may have affected task performance (*N* = 1). The final sample consisted of native English speakers who identified as 24% Asian, 11% Black, 59% Caucasian, and 4% mixed racial descent. Ten percent of the sample were of Hispanic descent. Two participants did not disclose information regarding their race or ethnicity. All participants provided written informed consent and received course credit for their participation. All procedures were approved by the Institutional Review Board at Emory University.

The current sample was collected as part of a prior investigation that tested hypotheses distinct from those examined here. Behavioral memory integration performance in the current sample (Varga and Bauer, 2017b; Varga et al., 2019a) and correlations with event-related potentials (ERPs) in a subset of the current participants (Varga and Bauer, 2017a) have been reported elsewhere. However, the present research features novel analyses of the systems-level oscillatory dynamics underlying individual differences in memory integration during learning.

### 2.2. Stimuli

The stimuli were 30 pairs of individual facts (i.e., stem facts) that could be combined to derive 30 novel integration facts (Bliss numbers S001–S030; see Bauer 2020). The facts were 4–10 words in length and spanned a range of educationally relevant domains including science, history, linguistics, and art. Prior research has shown that the facts are novel to adults, such that exposure to both stem facts from a pair was necessary to derive the target integration facts (Varga and Bauer, 2017b; Experiment 1). Notably, for one fact pair, successful derivation of the novel integration fact was equivalent with or without exposure to both individual stem facts (Varga and Bauer, 2017b; Experiment 1). Data corresponding to this stem fact pair was thus excluded from all reported analyses, resulting in a final stimulus set of 29 pairs of facts. Stimuli were presented using E-Prime 2.0 software (Psychology Software Tools, Pittsburgh, PA).

#### 2.2.1. Procedure

During the learning phase, EEG was recorded while participants learned 60 individual stem facts (30 pairs). To minimize saccadic eye movements during EEG acquisition, facts were presented one word at a time for 400 ms each (Fig. 1). Separate yet related facts from a to-be-integrated pair were separated by two to four intervening facts. This lag served to ensure temporal distance between related stem facts, thus necessitating retrieval of the prior, related fact upon encoding of a related fact. Related facts shared the same sentence-final word, which could cue participants to the overlapping relation. The EEG recording epoch encompassed the final word of each fact in addition to a 2000 ms blank screen following the offset of the final 400-ms relational word. The onset of a subsequent decision screen marked the end of the 2400 ms recording epoch, in which participants were instructed to indicate via a button-press response whether the fact was novel or known prior to participation in the study. The distractor task was intended to ensure attention to the content of the individual facts but was not considered in further data analysis. At no time were participants informed about the possibility to integrate separate yet related stem facts. The learning phase lasted approximately 10 min.

Following a delay of 5–10 min in which participants completed questionnaires, participants were tested for self-derivation of the 30 possible integration facts. As depicted in Fig. 1, the questions were presented in the form of incomplete sentences and participants were instructed to complete the final word of each fact. The target answer was always a common word, which served to reduce the impact of vocabulary differences on successful knowledge generation. Participants were given an unlimited amount of time to provide a response. Responses were scored online by the experimenter. Notably, some integration questions could elicit an open-ended response derived from the stem facts but that did not require integration of the previously learned stem facts. In these cases, participants were probed for an additional response (i.e., “Can you tell me another word that would also accurately complete this sentence?”). Once an answer was provided, the experimenter initiated presentation of the next question. EEG was acquired during the test phase but is not considered here. The test phase lasted approximately 15 min.

### 2.3. EEG data acquisition and pre-processing

Electrophysiological data was measured via 32 Ag/AgCl active sensors positioned in a fabric cap according to the 10–20 system (ActiCAP GmbH; Brain Products, Gilching, Germany). Reference electrodes were directly placed on the left and right mastoid bones while a ground electrode was placed on the center of the forehead. Conductive gel was applied to electrode contacts until impedances were lowered below 35 k Ω and typically below 15 k Ω. The EEG was sampled at 500 Hz using Pycorder software (Brain Products, Gilching, Germany) while participants were seated 90 cm in front of an LCD monitor. No filters were applied to the raw data during acquisition.

Before conducting any analyses, the raw electrophysiological data were pre-processed using EEGLAB 13.2.3b (Delorme and Makeig, 2004) and ERPLAB 4.0.3.1 (www.erplab.org) operating in Matlab R2014a (MathWorks, Natick, MA). To attenuate low and high frequency noise prior to artifact correction, the data were first bandpass filtered with a non-causal Butterworth filter with a half-amplitude cutoff of 0.1–30 Hz and a roll-off of 24 decibels/octave. To mitigate the potential influence of oculomotor artifacts on the oscillatory results, eye-blinks and saccades were detected and removed from the continuous EEG data using the standard *runica extended* Infomax independent component analysis (ICA) algorithm. To maximize the linear decomposition performance, segments of EEG data that contained excessive noise were manually scrubbed prior to running ICA and filtering. During manual scrubbing, only segments of data outside of the target recording and baseline epochs were removed, thus ensuring that the subsequently ICA-ed data and final analyses included the same number of trials across participants. Following ICA decomposition, components that accounted for eye activity were manually rejected based on careful examination of the component scalp map and time course.

### 2.4. Quantification and statistical analysis

#### 2.4.1. Overview

All electrophysiological analyses focused on initial fact learning, specifically on the 2.4-s recording epoch that started with presentation of the final word in each sentence (see Fig. 1). The recording epoch ended with the presentation of a visual prompt that cued participants to indicate whether they knew the fact prior to participating in the present study. The baseline was the 2.4-s period that immediately preceded the recording epoch. All electrophysiological data were analyzed in 0.8-s windows to minimize the complication of potential nonstationarity of the data in longer time windows (Mitra and Pesaran, 1999). For analyses of time periods longer than 0.8 s (i.e., the 2.4-s recording epoch), results were obtained by averaging across non-overlapping 0.8-s windows. The 0.8-s window size was chosen as a suitable duration into which to partition the 2.4-s recording epoch, enabling three equal time intervals while also allowing for multiple cycles of low frequency (namely, delta) oscillations in each window.

Analyses focused on a frequency range from 1 to 25 Hz for two reasons. First, to minimize misinterpretation of broadband artifacts, our analyses stipulated observation of oscillatory peaks in power spectrograms, which in our data were only observed in the alpha (9–12 Hz) range, or peaks in the Granger Causality spectrograms, which in our data were observed in the delta (1.5–3.5 Hz) and alpha (9–12 Hz) ranges. Broadband changes in oscillatory activity can result from aperiodic activity in the EEG signal, yet stipulation of clear spectral peaks in a predefined frequency band mitigates the likelihood of this misinterpretation (Buzsáki et al., 2012). Second, and consistent with the spectrograms analyzed here, previous scalp-based studies have indicated that frequencies above 25 Hz are contaminated by prominent higher-frequency muscle-related electrical artifacts (i.e., EMG) (Whitham et al., 2007) which may be misinterpreted as changes in oscillatory activity.

Unless otherwise noted, mean was reported and/or plotted as the measure of central tendency, and standard error of the mean was reported and/or plotted as the measure of precision. All analyses were conducted in Matlab 2020a (MathWorks, Natick MA) using publicly-available toolboxes and algorithms. These analyses are described in detail below and are followed by a section that describes the random permutation procedures used for evaluating statistical significance of electrophysiological data.

#### 2.4.2. Power and coherence

Whereas the role of oscillations in associative memory has typically been limited to examination of the magnitude of activity within individual channels, such as assessed through measures of oscillatory power (e.g., Jiang et al., 2020; Staresina et al., 2016), here we build on and extend upon this work by additionally examining the degree of *synchrony* between channels, as assessed through oscillatory coherence. Because oscillatory coherence measures the degree to which cortical regions synchronize with distal cortical regions, this measure enables a direct test of whether enhanced cortical integration between discrete neuronal ensembles is associated with enhanced knowledge integration.

Analyses of oscillatory power from individual channels and coherence between channels were conducted with Chronux version 2.12 (chronux.org), a publicly-available library of functions that implemented a multitaper fast Fourier transform (FFT) method (Bokil et al., 2010). Coherence was calculated as the absolute magnitude of coherency, which is cross spectrum normalized by the square root of the product of the two auto-spectra (i.e., power for each channel). A frequency bandwidth of ± 1.875 Hz was used, which enabled the use of 2 orthogonal tapers (windowing functions) for a time window of 0.8 s. In order to permit valid statistical comparisons between conditions, coherence estimates were Fisher Z transformed, power estimates were log 10 transformed (and multiplied by 10 to convert from bels to decibels), and both were corrected for bias as described previously (Bokil et al., 2007).

To quantify a network-level representation of synchrony, coherence was calculated for each pair of channels for each third of the 2.4-s recording epoch and baseline period. The baseline-subtracted coherence was then used as one measure of channel-channel pairwise oscillatory synchrony. These changes in pairwise coherence (“path weight”) were then summed for each electrode (“channel weight”) to evaluate whether that electrode became more or less synchronized with other electrodes across the cortex during thirds of the recording epoch relative to baseline (channel weight has sometimes been called node strength as a more general term; Rubinov and Sporns, 2010).

#### 2.4.3. Granger causality

Because delta peaks were not obvious in power spectra (see results), but delta oscillations have been previously implicated in large-scale cortical integration (Helfrich et al., 2017), we used Granger causality (GC) to verify that delta oscillations were present in the EEG signal. Granger causality quantifies channel-wise oscillatory patterns across different timepoints, thus discounting the influence of simultaneous signals, such as those generated by artifacts or volume conduction. More specifically, Granger Causality provides a directional estimate of the extent to which current oscillations in one channel predict future oscillations in a second channel (X _current →_Y _future_), above and beyond the predictiveness of the current oscillations in the second channel (Y _current →_Y _future_). If channel-wise GC methods reveal peaks in the spectrogram, such patterns provide another means of validating the presence of true oscillations in a given frequency band.

Granger causality was calculated using the publicly-available (users.sussex.ac.uk/∼lionelb/MVGC) Multivariate Granger Causality (MVGC) library of functions (Barnett and Seth, 2014). For the present study, data for each 0.8 s third of the recording epoch (and for thirds of the baseline) were analyzed for each subject in a channel-channel pairwise (bivariate) manner to maintain consistency with the coherence analysis approach. An autoregressive linear model was fit (using the “LWR ”algorithm) to the EEG data from each pair of channels from multiple prior time points to predict the data at the current time point. The present analysis first downsampled the EEG data to a sampling rate of 50 Hz and used 30 time points in the model, parameters selected based on the effort to validate delta frequency activity. An autocovariance sequence was calculated from the coefficients of this two-channel model, and spectral Granger causality was calculated from the autocovariance sequence (Barnett and Seth, 2014).

#### 2.4.4. Phase-amplitude cross-frequency modulation

Finally, to investigate the potential role of hierarchical, cross-frequency interactions in this form of integrative learning, we conducted cross-frequency coupling analyses. Guided by clear delta and alpha peaks in the Granger Causality spectrograms (see Fig. S3) as well as by previous work (Helfrich et al., 2017), we calculated the extent to which the phase of delta oscillations modulated the amplitude of alpha oscillations similar to that detailed by Tort et al. (2010). First, the normalized alpha amplitude was calculated as a function of delta phase. Specifically, for each channel, the electrophysiological data for each participant were re-filtered (using a zero-phase digital band-pass filter; “filtfilt”function in Matlab) separately into delta (1.5–3.5 Hz) and alpha (9–12 Hz) bands. Delta phase was estimated for each sample by calculating the phase angle of the Hilbert-transformed delta band. Alpha amplitude was estimated for each sample by calculating the complex magnitude of the Hilbert-transformed alpha band. For each channel and trial, alpha amplitudes were binned by 20°bins of delta phase and were then averaged within each phase bin separately for each 0.8-s third of the recording epoch and of the baseline. A channel-wise Modulation Index (MI) was obtained by calculating the dissimilarity between this distribution of normalized alpha amplitudes across delta phase bins to a control comparison distribution using Kullback–Leibler distance as the metric of dissimilarity. Tort et al. (2010) used a uniform (flat) distribution as the comparison distribution. Here, to address the possibility of spurious estimates resulting from volume conduction of low frequency oscillations or electrical artifacts, we used a different comparison distribution of alpha amplitude across delta phase bins. Specifically, for the comparison distribution, instead of using the delta signal for each channel, we used a delta signal obtained by taking the grand mean of all channels’ delta-filtered EEG. We then used the distribution of an individual channel’s normalized alpha amplitudes across delta phase bins of that grand mean signal as the comparison distribution. Thus, a higher MI corresponded to more modulation of alpha amplitude by delta phase above and beyond that which would be expected to result from volume conduction of low-frequency oscillations or electrical artifacts. As these MI values already represented a difference score (a metric of distribution dissimilarity), no further baseline subtraction was used.

#### 2.4.5. Calculation of statistical significance

A cluster-based random permutation approach was used to evaluate the statistical significance of all electrophysiological contrasts. The approach was based on prior reports (Maris et al., 2007b; Maris and Oostenveld, 2007a) and controlled for the family-wise error rate either across frequency bins or across channels, depending on the analysis. That is, the advantage of the cluster-based procedure is that it does not calculate statistical significance for each individual frequency (or channel, depending on the analysis) and thus does not inflate the overall alpha rate by performing multiple comparisons. Details for each analysis are provided below.

For power spectrogram differences between the fact recording epoch and baseline period, the original fact recording epoch and baseline power spectra for all participants was reshuffled 1000 times such that, in each shuffle, a participant’s fact recording epoch data was randomly labeled as coming from the fact recording epoch or baseline. The mean and standard deviation across participants of the fact-baseline difference was then calculated for the original data and for each random shuffle. For each electrode channel, an initial threshold of 0 ± 1 standard deviations (across participants) of the original difference was used to identify suprathreshold frequency clusters (frequency ranges) in both the original data and in each randomly shuffled data set. Specifically, for each random shuffle, the largest frequency cluster was identified (largest summed absolute value of the within-cluster power difference), establishing a distribution of the largest random cluster sums. For the original data, only clusters whose sum exceeded the 99th percentile value of this random distribution were identified as statistically significant (i.e., two-tailed *p* < 0.01). A relatively conservative *p* value of 0.01 was used because this approach for evaluating power differences accounted for multiple comparisons across frequency bins but not across channels.

A similar cluster-based random permutation approach was used to evaluate the statistical significance of channel weights of coherence differences between thirds of the fact recording epoch and baseline period, an approach used previously to evaluate patterns of EEG oscillations (Manns et al., 2018). For channel weights, clusters were operationalized as neighboring channels rather than neighboring frequency bins. Specifically, each participant’s data were randomly reshuffled 1000 times between fact recording epoch and baseline. For the original data and for each random shuffle, the mean difference (across participants) in channel weights between cue and fixation periods was calculated. Neighboring channels for which the channel weight exceeded ± 0.5 standard deviations (across participants) of the difference were assigned to a cluster. The absolute value of the within-cluster sum was calculated for every cluster, and the maximum cluster value was obtained for every random permutation. For the observed data, only clusters the sum of which exceeded the 95th percentile value of these random cluster values were identified as being statistically significant (i.e., two-tailed *p* < 0.05). An initial cluster threshold of 0.5 standard deviations was used (rather than 1.0 used for frequency clusters for power) because these clusters were based on the spatial layout of the electrode contacts over the scalp. A p value of 0.05 (or 0.05/3) was used (rather than 0.01) because the channel weight cluster approach used only one frequency range (9–12 Hz) and did not involve making comparisons separately for each channel, as the power analysis did. Effect sizes for significant channel clusters were calculated as Cohen’s d by dividing the mean difference by the standard deviation of the difference for within-subjects contrasts or by dividing the difference of group means by the pooled standard deviation for between-group contrasts. The same channel-cluster random permutation approach used for evaluating contrasts involving alpha coherence channel weights was also used for contrasts involving the delta-alpha phase-amplitude modulation indices for each channel.

## 3. Results

**Fig. 2.**
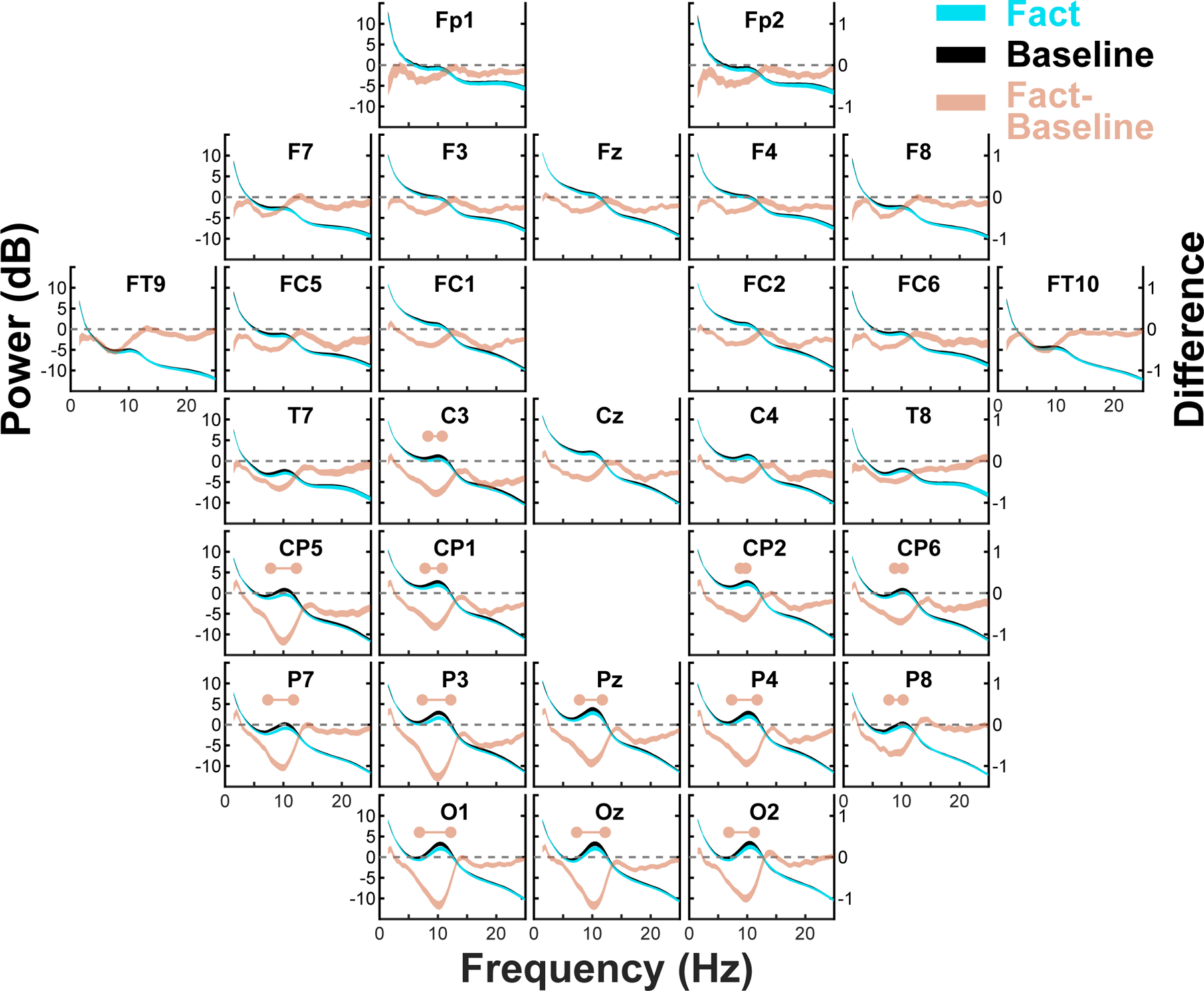
Oscillations in the alpha (9–12 Hz) range were prominent across all 30 recording channels during the 2.4 s fact recording epoch and baseline period (the 2.4 s preceding the recoding epoch). The blue and black lines show mean (center of line) and SEM (thickness of line above and below mean) power across all 80 participants for the fact epoch and baseline period, respectively, and are plotted against the left Y axes (absolute power in dB) and X axes (frequency in Hz). The peach lines show for each channel the fact-baseline differences (mean ± SEM across participants) and are plotted against the right Y axes (power difference in dB) and X axes. Dashed horizontal lines indicate 0 (no difference) on the right Y axes. Horizontal peach lines bookended by circles indicate frequency ranges for which the difference was statistically significant (*p* < .01; see Section 2.4.5 [Calculation of statistical significance] for details of significance testing). Statistically significant power decreases encompassing the alpha range (9–12 Hz) were observed in numerous central and posterior channels. No statistically significant power increases in the delta range (1.5–3.5 Hz) were observed.

Alpha oscillations were prominent in EEG recordings as participants learned the pairs of facts, each pair conceptually connected by a matching final word but temporally separated by intervening facts (Fig. 1). Fig. 2 shows power spectrograms for each of the 30 recording channels and focuses on the 2.4-s recoding epoch that started with the presentation of the final word in each sentence. The plots show alpha power (dB) peaks at approximately 10.5 Hz for both the fact recording epoch and the immediately preceding 2.4-s baseline period, which were more prominent for posterior relative to anterior channels. The plots also show for each channel differences in power between the fact and baseline periods. Power in the alpha frequency range (9–12 Hz) showed statistically significant (p < .01) reductions during the fact recording epoch for thirteen mostly posterior channels (Oz, O1/2, Pz, P3/4, P7/8, CP1/2, CP5/6, and C3). Thus, the data suggest that these alpha power shifts correspond to event-related changes in neuronal oscillations during fact learning. Statistically significant effects in power were not observed in the delta frequency range (1.5–3.5 Hz). Thus, our oscillatory analyses focused first on alpha coherence before revisiting delta oscillations with additional analytic techniques that could further verify the presence of delta oscillations in the EEG signal (see Granger Causality below).

**Fig. 3.**
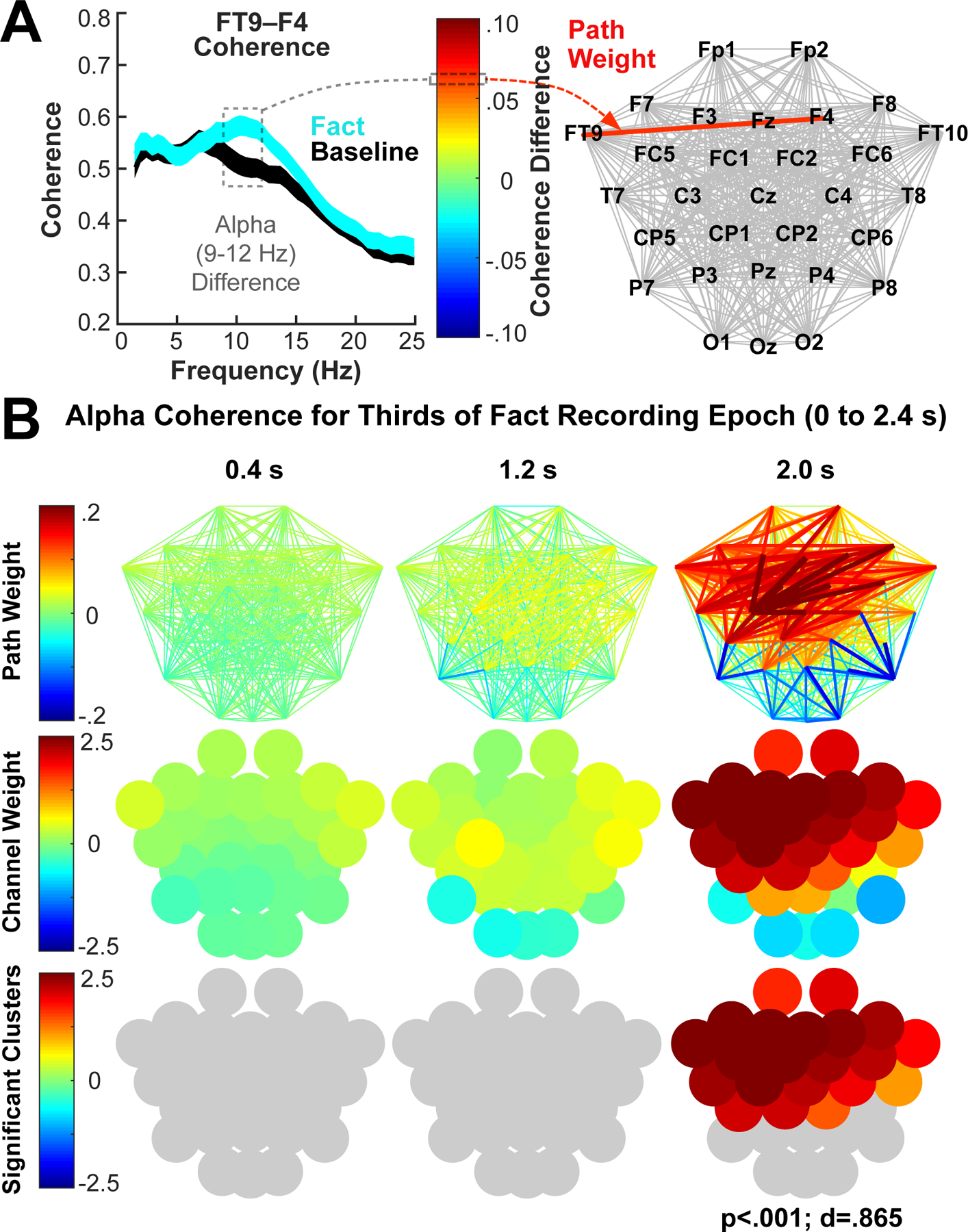
Analyses of channel-channel alpha coherence indicated that alpha coherence during the fact recording epoch significantly increased relative to baseline across a large cluster of channels. (A) Depiction of alpha coherence analyses using one example pair of channels (FT9 and F4; fact and baseline lines show mean ± SEM across 80 participants). The mean fact-baseline coherence difference across participants in the alpha (9–12 Hz) range was plotted as a color-coded path weight on a network of all possible paths between all 30 channels. (B) Plots of alpha coherence path weights and channel weights averaged across fact condition (Fact 1 and Fact 2) but separately for thirds (0.8-s time intervals indicated by the center-point) of the 2.4 s fact recording epoch. The weights for all paths connected to a channel were summed to calculate the weight for that channel, and the channel weights were plotted as color-coded circles according to channel position. Statistical significance relative to baseline was evaluated using a cluster-based (neighboring channels) random permutation approach (see Section 2.4.5 [Calculation of statistical significance] for details of significance testing). P- and d-values denote statistical significance and effect size (Cohen’s d), respectively.

Based on the observed changes in alpha power from individual channels, the data were next analyzed for possible task-related increases in alpha synchrony *between* recording channels. As an estimate of cortical synchrony, we calculated event-related (Fact-Baseline) alpha coherence between two channels, which we referred to as a path weight. Fig. 3A illustrates the approach for the 2.4-s recording epoch separated into 0.8-s time windows, which permitted examination of dynamical changes in coherence over the course of fact processing. Specifically, we summed the 29 path weights connected to each channel to calculate a channel weight, which was used as an estimate of the extent to which each channel showed a change in alpha coherence during the fact recording epoch relative to baseline. The results indicated that alpha coherence increased markedly between many channels overlaying frontal, temporal, and parietal regions during the last 0.8-s of the recording epoch (Fig. 3B).

**Fig. 4.**
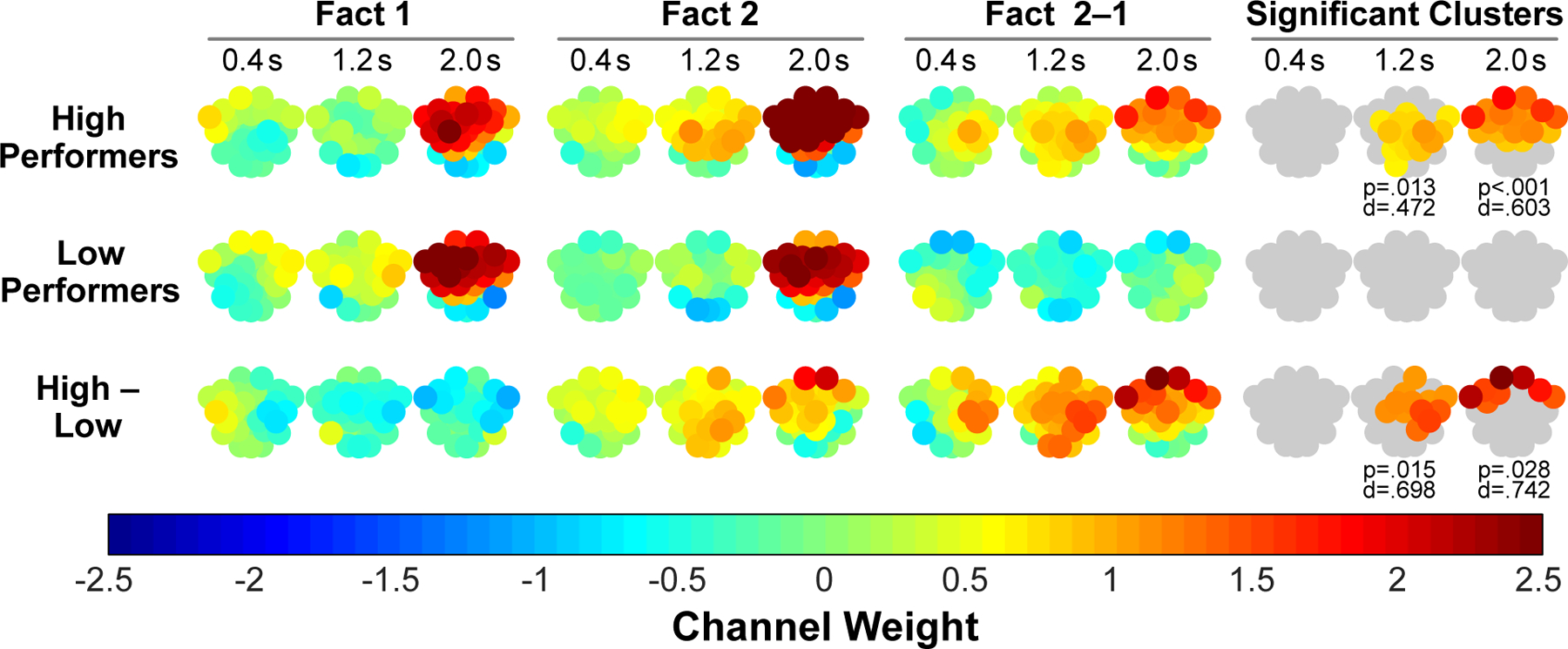
High performers showed more alpha coherence for the second fact (i.e., the only opportunity to integrate the facts) versus the first fact in each pair of related facts, whereas low performers did not. Increased alpha coherence in high performers was first evident at posterior central-parietal channels and subsequently extended to frontal regions. Alpha coherence channel weights are plotted for thirds (0.8 s time intervals indicated by the center-point) of the 2.4 s recording epoch for the first (Fact 1) and second (Fact 2) paired facts and are shown separately for high and low performers. Statistical significance of differences between Fact 2 and Fact 1 and between high and low performers was evaluated using a cluster-based (neighboring channels) random permutation approach (Section 2.4.5 [Calculation of statistical significance] for details of significance testing). P- and d-values denote statistical significance and effect size (Cohen’s d), respectively.

We next examined whether alpha coherence increased during processing of a related fact (Fact 2) relative to processing an unrelated fact (Fact 1), providing a within-subject index of changes in cortical integration during opportunities for fact integration. Accordingly, Fig. 4 shows plots of alpha coherence channel weights separately for Fact 1 and Fact 2 during learning. If alpha coherence tracks integration of current and reactivated knowledge during related events, we further reasoned that enhanced coherence should be more evident in participants who performed in the top half (40 high performers) relative to the bottom half (40 low performers) on the subsequent test for knowledge integration (Fig. 1). Indeed, the interaction between fact condition (Fact 2 minus Fact 1) and integration performance (high performers minus low performers) was statistically significant, indicating that high performers showed disproportionately increased alpha coherence for Fact 2 (Fig. 4). Examination of changes in patterns of coherence across 0.8-s thirds of the recording epoch provided further insight into the unfolding spatiotemporal dynamics, revealing increases over occipital, parietal, and temporal sites by around 1.2 s that then shifted to frontal sites by around 2.0 s. Parallel analyses of alpha power revealed no effect of fact condition or subsequent performance (Fig. S1), suggesting that changes in oscillatory coherence were not attributable to simple changes in oscillatory power, nor was oscillatory power a sensitive index of either within- or between-subject differences in memory integration. Furthermore, when we split participants by working memory performance, the pattern of coherence was rather different (Fig. S2). In particular, enhanced alpha coherence was no longer observed for Fact 2 in high performers, suggesting that the pattern of coherence in Fig. 4 is unlikely to reflect more general factors, such as differences in working memory capacity between participants and/or differences in cognitive load between Fact 1 and Fact 2.

Having demonstrated that enhanced alpha synchrony during related events was related to better integration performance, we next asked if interactions between delta and alpha oscillations might further relate to fact integration performance. Because delta oscillations were not apparent in the power spectra (Fig. 2), it was first necessary to verify the presence of delta in the EEG recordings. To that end, we further explored Granger Causality (GC) between pairs of channels as a function of oscillatory frequency, a metric that discounts simultaneous EEG changes and thus discounts the influence of potential electrical artifacts and volume conduction on detection of oscillatory activity. Fig. S3 shows, for each channel and frequency, mean GC for that channel and all other channels, separately for GC outwardly directed away from a channel and inwardly directed to a channel, reflecting the degree to which that channel predicts (“Out”) and is predicted by (“In”) other channels, respectively (see Materials and Methods). In addition to clear alpha peaks at approximately 10.5 Hz, the plots showed clear delta peaks at approximately 2 Hz, indicating that delta oscillations were a prominent component of the EEG signal.

**Fig. 5.**
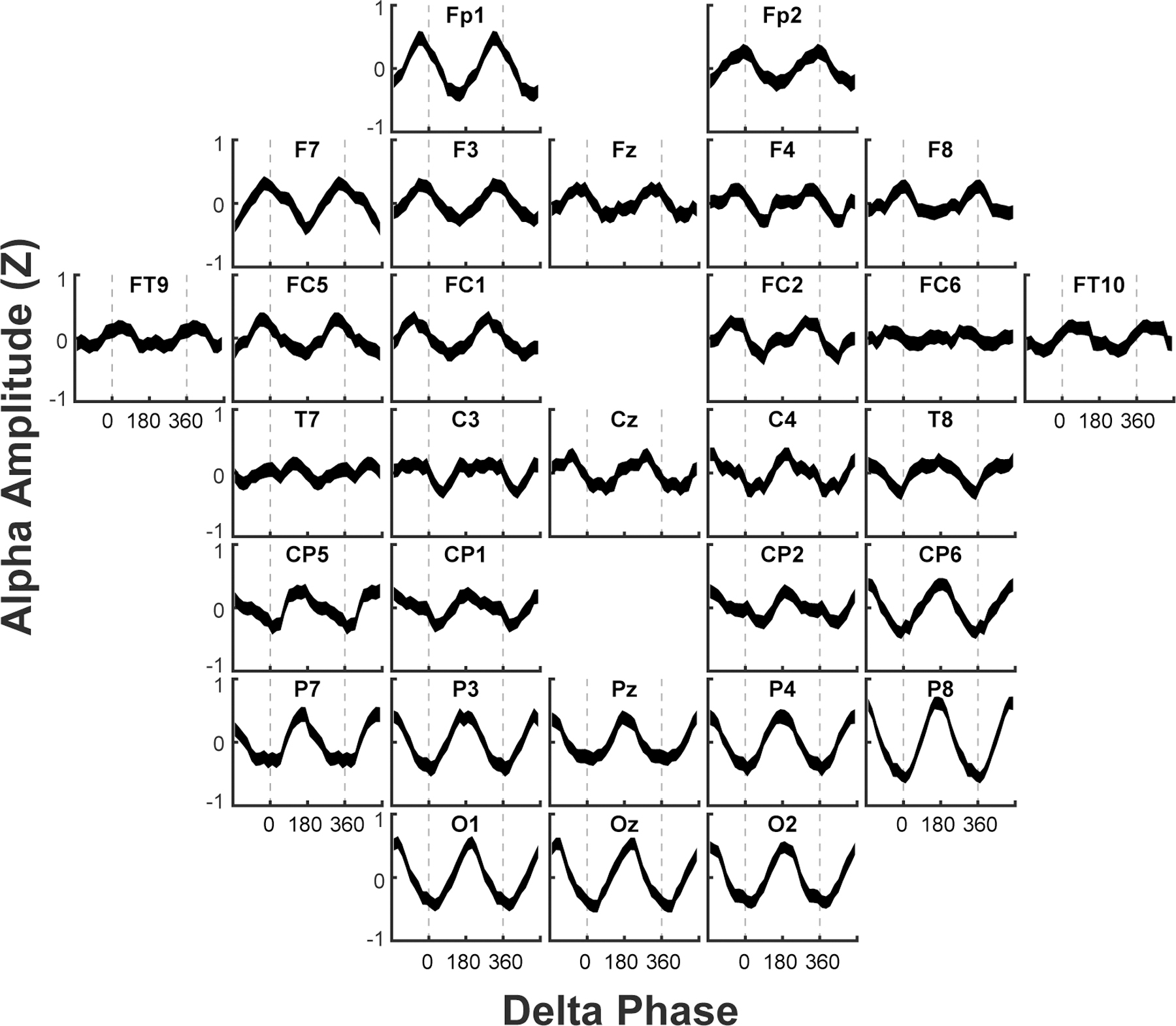
The phase of delta oscillations strongly modulated the amplitude of alpha oscillations, particularly at posterior sites. The mean (± SEM) standardized (Z-scored) alpha amplitude during the fact epoch is plotted for each channel as a function of that channel’s delta phase (in degrees; two full phase cycles are plotted for better visibility of the patterns). The vertical dashed lines indicate the phase of the delta peak (0°and 360°), with one complete cycle spanning 0–360°. Modulation of alpha amplitude by delta phase is reflected by sinusoidal waveforms, indicating how increases and decreases in alpha power occur rhythmically in accord with the relative delta phase. For example, for channel Oz, increases in alpha power occur near the delta trough (180°) and decreases in alpha power occur near the delta peak (0 and 360°).

**Fig. 6.**
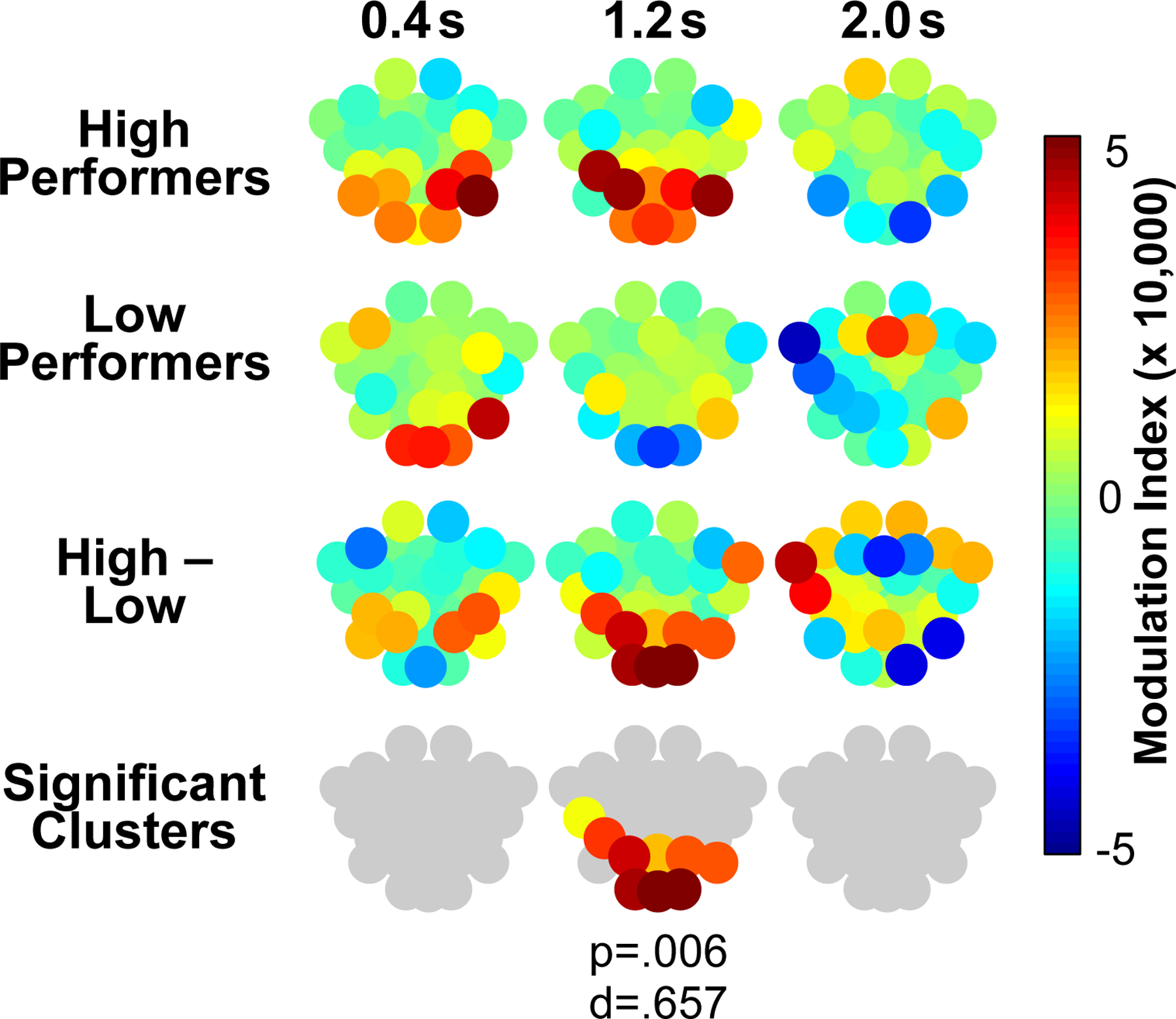
The extent to which channel-wise delta phase modulated channel-wise alpha amplitude during 0.8 s thirds of the fact epoch (relative to a grand mean delta signal computed across all channels as a baseline comparison) is shown for each channel for high and low performers as a color-coded modulation index (see Section 2.4.4 [Phase-amplitude cross-frequency modulation] for details regarding how the modulation index was calculated). For the high and low performers, positive numbers indicate increased delta-alpha modulation during the fact epoch relative to a baseline that accounted for possible volume-conduction artifacts. For high-low differences, positive numbers indicate greater delta-alpha modulation during the fact epoch (relative to the baseline) for high performers. Statistical significance of differences between high and low performers was evaluated using a cluster-based (neighboring channels) random permutation approach (see Section 2.4.5 [Calculation of statistical significance] for details of significance testing). P- and d-values denote statistical significance and effect size (Cohen’s d), respectively.

Based on the verification of prominent delta in addition to alpha oscillations in the EEG recordings, we next asked if the amplitude of the faster alpha oscillations was modulated by the slower delta wave, a type of phase-amplitude CFC that has previously been implicated in context-dependent perception (Helfrich et al., 2017). Fig. 5 shows that the amplitude of alpha oscillations showed clear modulation by delta phase for most channels, a depth of modulation (as calculated by a Modulation Index; see section 2.4.4 [Phase-amplitude cross-frequency modulation]) that was prominent in posterior channels during the fact recording epoch. Changes in delta-alpha phase-amplitude modulation were pronounced for only the high performers, and Fig. 6 also shows that the difference between high performers and low performers was statistically significant in the final fact recording window (0.05 rather than 0.05/3 when using a cluster-based random permutation approach). Like findings of alpha coherence, high performers’ delta-alpha phase-amplitude modulation did not differ significantly as a function of domain-general factors such as working memory capacity (Fig. S4). Analysis of delta-alpha CFC further split by Fact 1 and Fact 2 further revealed no significant effects when we used our pre-defined cluster threshold (i.e., only channels that exceeded 1 SD of the difference), but at a threshold that only admitted channels exhibiting larger effects (i.e., *>* 2 SD), there was evidence that enhanced posterior CFC in high performers was specific to Fact 2 (Fig. S5). Thus, the timing and topography of delta-alpha CFC complements evidence of enhanced posterior alpha coherence for Fact 2 in high performers at around 1.2 s in the recording epoch, both of which were followed by enhanced frontal alpha coherence at around 2.0 s in the recording epoch.

## 4. Discussion

The present results from a fact integration task provided key insights about the role of cortical alpha synchrony and delta-alpha cross-frequency coupling (CFC) in enabling memory integration by linking separate yet related cortical ensembles. Participants’ behavior on a test of new knowledge generation through fact integration exhibited a wide range in performance (Fig. 1), raising a key question of whether cortical alpha coherence and delta-alpha CFC during initial learning of to-be-integrated facts could explain variability in generation of the novel integration facts. During fact learning, EEG recordings indicated that, relative to participants who showed lower fact integration performance, high performers showed an early (1.2 s) increase in central-posterior cortical alpha synchrony followed by a later (2.0 s) increase in frontal alpha synchrony (Fig. 4). Moreover, for high performers only, this pattern of alpha synchrony was significantly higher when they read the second fact versus the first fact in a related pair, connecting the alpha synchrony to the opportunity to integrate the second fact with memory of the first fact (Fig. 4). High performers, but not low performers, also showed modulation of posterior alpha amplitude by delta phase, a CFC pattern that temporally and spatially coincided with early (1.2 s) increases in alpha coherence (Fig. 6).

One possible interpretation is that, particularly for participants who subsequently performed well on the integration test, reading the first fact engaged semantic processing, and reading the second fact did the same and additionally triggered memory and semantic processing of the first. This interpretation presumes that semantic processing of both current and reactivated knowledge would benefit from the precise temporal coordination of cortex-wide networks of neurons whose activity is thought to instantiate the knowledge, the type of temporal coordination enabled by oscillatory synchrony and cross-frequency coupling. By this view of higher performers’ oscillatory patterns, the early (1.2 s) increases in posterior alpha coherence, particularly for the second fact, scaled with the amount of activated information (two facts *>* one fact), whereas the early (1.2 s) increases in posterior delta-alpha CFC corresponded more generally with semantic processing, as even processing of individual facts (fact 1 or fact 2) entails simultaneous coordination of multiple cortical traces. Moreover, the later (2.0 s) increases in anterior alpha coherence, particularly for the second facts, could be interpreted as increased frontal-mediated processes more specifically related to knowledge integration. We discuss below the implications of the results for our understanding of how new information is dynamically integrated with distributed cortical knowledge —a critical step in constructing a knowledge base.

### 4.1. Alpha coherence as a measure of functional integration of distributed cortical regions

Complex cognitive functions, including memory integration, are supported by functional networks rather than single brain regions. For example, prior fMRI studies have demonstrated that memory integration is supported by multiple cortical and subcortical regions (Zeithamova and Preston, 2010), and that enhanced connectivity between regions predicts successful performance (Zeithamova et al., 2012). Although such data offer important clues regarding the specific anatomical regions that functionally interact, techniques that offer millisecond-level sampling resolution are needed to begin to understand how information is transferred and linked between them. One view is that, when oscillations among neuronal ensembles synchronize, the congruence of excitatory peaks and inhibitory troughs increases the probability that information leaving one area will arrive when the other area is maximally excitable (Fries, 2005; Palva and Palva, 2011; Varela et al., 2001). Electrophysiological studies in rodents (Buzsáki and Draguhn, 2004; Place et al., 2016; Tort et al., 2009) and magnetoencephalography data in humans (Backus et al., 2016) support this idea, demonstrating that oscillatory synchrony promotes routing and integration of information across the brain during learning. Here, we leveraged this analytic approach to test whether differences in oscillatory synchrony relate to differences in knowledge integration, thus providing important insight into how separate yet related knowledge is spontaneously integrated during learning.

The first step in the analytic approach was to validate that the scalp-based alpha coherence metric was sensitive to changes in cortical integration in all participants by quantifying for each channel the change in alpha coherence with other channels during processing of new semantic facts (Fig. 3). Alpha oscillations have been shown to correlate with a number of specific cognitive processes (Klimesch, 2012; Palva and Palva, 2007, 2011). Yet there is a growing consensus that they constitute a broader neurophysiological basis for large-scale cortical integration, relating generally to temporal coordination between adjacent and non-adjacent cortical areas (Fries, 2005; Palva and Palva, 2011; Sadaghiani and Kleinschmidt, 2016; Varela et al., 2001). For example, evidence from nonhuman animal studies suggests that alpha oscillations reflect both cortical-cortical and cortical-thalamic interactions (Bollimunta et al., 2008, 2011; Saalmann et al., 2012). Prior fMRI research has shown that left temporal cortices and prefrontal cortex (PFC) are recruited together during semantic processing (Badre et al., 2005; Thomspon-Schill et al., 1999). We thus expected to observe increased frontotemporal alpha coherence following the final word of each fact, which enabled participants to integrate the preceding words to generate a sentence-level representation. Indeed, across both participant groups (high and low performers) and fact conditions (Fact 1 and Fact 2), we observed strong alpha coherence above left temporal and frontal electrodes about 2 s following the sentence-final word (Fig. 3), verifying that changes in coupling of task-relevant functional networks–reflecting cortical integration–could be observed across all participants and conditions. The overall frontotemporal coherence pattern is consistent with participants forming an integrated representation of the individual sentences.

### 4.2. Alpha coherence indexed retrieval and integration of distributed cortical knowledge

A key question was whether reading the second fact in a pair of related facts would reflect oscillatory correlates of fact integration in the cortex, a hypothesized mechanism for dynamically linking ensembles of information distributed across cortical nodes. By contrasting alpha coherence between pairs of novel facts that shared the same final word (e.g., *pips* in Fact 1 vs. Fact 2; Fig. 1), the current results revealed the oscillatory dynamics uniquely associated with integrating novel yet related information (Fact 2 *>* Fact 1). Previous ERP data that included a subset of the current sample indicated that knowledge integration entails multiple temporally-staged processes, including explicit comprehension of the meaning of to-be-integrated facts based on prior knowledge that is simultaneously reactivated (at about 1.2 s) followed by representation of the integrated relation between the new and reactivated knowledge (at about 1.6 s and later) (Varga and Bauer, 2017a). In the present study, we replicate and extend these findings to provide evidence for the role of alpha synchrony in activating and linking the discretely learned cortical traces to the same neuronal ensemble to form an integrated trace.

High performers, but not low performers on the fact integration test, showed increased posterior alpha coherence that was maximal over central-parietal sites at about 1.2 s for related relative to unrelated facts. Anatomical evidence reveals that parietal cortex exhibits connections to distributed cortical systems (Dehaene et al., 2006) and guides retrieval of abstract representations (Morton et al., 2020), suggesting that reactivation of cohesive, detailed memory representations would result from increased synchrony between parietal cortex and other cortical areas (Favila et al., 2018; Kuhl et al., 2013). Moreover, recent temporal evidence demonstrates that oscillatory correlates of explicit memory retrieval are evident approximately 1 and 2 s following a cue (Staresina et al., 2016; Varga & Bauer, 2017a). The timing and topography of this effect thus suggests that initially unfolding alpha coherence reflects reactivation of the prior, relevant semantic fact, and more specifically, synchronous recruitment of cortical traces stored across distributed semantic and sensory cortical regions (Barsalou, 2008; Huth et al., 2012; Martin, 2007; Polyn et al., 2005).

Once prior, relevant knowledge is cognitively available, alpha synchronization may then support integration of the newly and previously learned facts. Prevailing theoretical models presume that such memory integration depends on precisely timed coordination of distributed cortical ensembles that represent related memories (Kumaran and McClelland, 2012; Preston and Eichenbaum, 2013) and that prefrontal cortex modulates integration of the new and reactivated knowledge (Preston and Eichenbaum, 2013; Schlichting and Preston, 2015; Varga et al., 2021), through biasing new encoding toward behaviorally relevant memories (Mack et al., 2020; van Kesteren et al., 2014). However, relatively sparse empirical data has examined the precise mechanism through which PFC influences memory integration or the timing of its influence during new learning. Here, the results provide direct evidence that frontal alpha coherence supports knowledge integration during learning. In particular, enhanced frontal alpha coherence was observed during Fact 2 in participants who successfully generated the novel integration facts, a pattern that emerged at about 2 s, immediately following evidence of enhanced central-parietal alpha coherence. In addition to playing a specialized role in memory integration, PFC has been shown to maintain behaviorally relevant information over delays, even in the face of intervening information (Miller et al., 1996). Thus, whereas earlier posterior coherence may support simultaneous activation of newly and reactivated memories, frontal alpha coherence, in turn, may guide representation of the integrated relation between the new fact and reactivated knowledge, providing a means of linking new information to existing knowledge previously stored in neocortex.

### 4.3. Delta-alpha modulation improved temporal coordination of distributed cortical ensembles

In addition to enhanced alpha synchronization in high performers during processing of related facts, the results further showed that delta oscillations modulated alpha amplitude in a delta phase-dependent manner. That is, the magnitude of oscillations within the alpha band waxed and waned in accord with the ongoing delta phase, a pattern that was particularly pronounced at posterior sites early, at around 1.2 seconds. Theoretical models have proposed that such cross-frequency coupling constitutes a mechanism for coordinating faster, spike-based computations across large-scale brain networks (Canolty and Knight, 2010). In the present research, posterior delta-alpha CFC emerged at the same time as enhanced posterior alpha coherence implicated in explicit meaning comprehension and memory activation, suggesting that these simultaneous patterns may play complementary roles in coordinating distributed cortical modules that represent semantic knowledge. That is, because individual semantic concepts are represented by nodes distributed across the cortex (Barsalou, 2008; Huth et al., 2012; Martin, 2007), explicit activation of the current and/or reactivated fact requires precisely timed coordination of such distributed information to support unified cognitive interpretations (Helfrich and Knight, 2016). By this view, increased posterior delta-alpha CFC may reflect a general mechanism for regulating such widespread information integration, through improving the temporal coordination of alpha oscillations that enable synchronous recruitment of distributed traces.

Mechanistically, by modulating the amplitude of the faster alpha rhythm in accordance with the slower delta phase, such coordinated oscillations may provide additional means, beyond alpha synchrony alone, through which to promote spike timing, and in turn, synaptic transmission and plasticity across widely distributed neocortical traces. That is, timing the bursts of the faster alpha rhythm relative to the phase of the slower delta oscillation likely augmented precisely timed, simultaneous ensemble activation reflected by alpha synchronization. That modulation of alpha oscillations by delta phase coincided with evidence of enhanced alpha coherence in the same time window and spatial regions further supports this proposal. More specifically, activating and comprehending the meaning of the current and/or reactivated semantic fact, as reflected by enhanced posterior alpha coherence at 1.2 s, may have up-regulated the demand to simultaneously coordinate knowledge traces widely distributed throughout the cortex. Thus, if delta-alpha CFC provides a means of regulating such large-scale activation, strong, transient phase-amplitude coupling would be expected to emerge alongside such central-posterior alpha synchronization, as was the case in the present research.

Finally, the present results are consistent with prior views of cross-frequency coupling, which propose that slower, low-frequency activity can track internal cognitive processes and states associated with memory (Canolty and Knight, 2010). Thus, in addition to optimizing posterior alpha coherence that supports synaptic transmission necessary for activating the current and/or remembered fact, it is possible that such delta-alpha CFC also provided a mechanism for making the activated semantic contents cognitively accessible. In line with this proposal, we subsequently observed enhanced cortical alpha synchronization over frontal regions in high performers during processing of Fact 2, implying that this later alpha coherence signature may have corresponded to synaptic modification of previous knowledge to link the new fact following earlier meaning abstraction. Notably, when the CFC cluster threshold was adjusted to admit only channels that exhibited larger delta-alpha modulation (Fig. S5), we showed that this pattern was specific to the second, to-be-integrated facts in high performers, suggesting that this form of temporal coordination and cognitive accessibility may be particularly pronounced when prior knowledge is reactivated. Future research is needed to address the precise source of this cross-frequency engagement.

## 5. Conclusions

In summary, long-standing theories of cognition have argued that the complexity of one’s semantic knowledge base depends not only on the amount of individual facts accrued over time, but also on the degree to which that information is coherently linked and organized in memory (Chi and Koeske, 1983). The present results provide new evidence for how such integration may be achieved. We propose that early central-posterior alpha coherence and delta-alpha CFC play a key role in coordinating activation of distributed cortical knowledge traces, thereby enabling formation of and access to unified cognitive interpretations of current and reactivated knowledge. We further suggest that later frontal alpha coherence plays a specialized role in linking the current and reactivated knowledge to the same cortical ensemble, through biasing encoding of the currently available information toward the integrated relation to contextually relevant information that was made cognitively available through memory reactivation. The present results thus build on prevailing cognitive theories, revealing the coordinated neural dynamics through which long-lasting, interconnected knowledge structures are formed and deployed to guide complex cognitive behaviors. Understanding the neural underpinnings of variability in integrated knowledge representation also has important educational implications. In particular, striking variability in knowledge extension through integration predicts concurrent and longitudinal academic success in the same individuals tested here (Varga et al., 2019a, 2019b). By revealing neurophysiological markers that distinguish individuals who do or do not readily engage in integrative learning, the present work thus constitutes a cornerstone for advancing interventions designed to improve these functionally significant neuronal dynamics, and by extension, cognitive and academic behaviors that rely on flexible knowledge integration.

## Supplementary Material

1
