# Supplementary material for: Delta-modulated cortical alpha oscillations support new knowledge generation through memory integration
Source: Neuroimage. Author manuscript; Available in PMC 2022 Jan 28. (PMC8796818; doi:10.1016/j.neuroimage.2021.118600)
Supplement: 1 [file NIHMS1755359-supplement-1.pdf]

Supplemental Figure Captions

Figure S1. High performers and low performers both showed similar patterns of alpha power for the second fact versus the first fact in each pair of facts. Analyses of alpha (9-12 Hz) power as a function of condition (Fact 1 versus Fact 2) and performance (high versus low performers) revealed no significant differences. Mean baseline-subtracted alpha power (dB) is plotted for each channel for thirds (0.8-s time intervals indicated by the center-point) of the 2.4-s recording epoch for the first (Fact 1) and second (Fact 2) paired facts and are shown separately for high and low performers. Statistical significance of differences between Fact 2 and Fact 1 and between high and low performers was evaluated using a cluster-based (neighboring channels) random permutation approach similar to that used for evaluation of changes in coherence (see section 2.4.5 [Calculation of Statistical Significance] for details of significance testing).

Figure S2. Participants who scored low on a test of working memory (WM) showed more alpha coherence for the second fact versus the first fact in each pair of related facts, whereas high working memory performers did not. Alpha coherence channel weights are plotted for thirds (0.8-s time intervals indicated by the center-point) of the 2.4-s recording epoch for the first (Fact 1) and second (Fact 2) paired facts and are shown separately for high and low working memory performers. Importantly, for participants with higher working memory capacity who were disproportionately more likely to perform well on the knowledge integration task, alpha coherence during the fact epoch (relative to baseline) was not shown to differ between Fact 1 and Fact 2, suggesting that general differences in cognitive load between fact types were unlikely to account for the pattern of results in main Figure 4. Statistical significance of differences between Fact 2 and Fact 1 and between high and low working memory performers was evaluated using a

cluster-based (neighboring channels) random permutation approach (see section 2.4.5 [Calculation of Statistical Significance] for details of significance testing). P- and d-values denote statistical significance and effect size (Cohen's d), respectively.

Figure S3. Analyses of granger causality revealed clear oscillatory peaks in both the delta (1.5-3.5 Hz) range and alpha (9-12 Hz) range across all 30 recording channels. Plots of granger causality as a function of frequency during the fact epoch for each channel (mean ± SEM across 80 participants). The lines for each channel show the mean granger causality between that channel and all other channels and are plotted separately for outwardly directed granger causality (Out; e.g., channel F4 → FT9) and inwardly directed granger causality (In, e.g., F4 ← FT9).

Figure S4. Delta-alpha modulation indices were not significantly different between individuals with high versus low working memory capacity. The extent to which delta phase modulated alpha amplitude during 0.8-s thirds of the fact epoch (relative to baseline) is shown for each channel for individuals with high and low working memory capacity as a color-coded modulation index (see section 2.4.4 [Phase-amplitude cross-frequency modulation] for details regarding how the modulation index was calculated). For the high and low working memory groups, positive numbers indicate increased delta-alpha modulation during the fact epoch relative to baseline. For high-low differences, delta-alpha modulation during the fact epoch (relative to baseline) was not shown to differ as a function of general cognitive differences between groups. Statistical significance of differences between high and low working memory groups was evaluated using a cluster-based (neighboring channels) random permutation approach (see section 2.4.5 [Calculation of Statistical Significance] for details of significance testing).

Figure S5. High performers showed more posterior delta-alpha modulation for second versus the first fact, whereas low performers did not, at least when examined at a cluster threshold that admitted channels exhibiting larger effects (see Method section 2.4.5 [Calculation of Statistical Significance] and Results for details). The extent to which channel-wise delta phase modulated channel-wise alpha amplitude during 0.8-s thirds of the fact epoch (relative to a grand mean delta signal computed across all channels as a baseline comparison) is shown for each channel as a color-coded modulation index, shown separately for the first (Fact 1) and second (Fact 2) paired facts and for high and low performers (see section 2.4.4 [Phase-amplitude cross-frequency modulation] for details regarding how the modulation index was calculated). For the high and low performers, positive numbers indicate increased delta-alpha modulation during the fact epoch relative to a baseline that accounted for possible volume-conduction artifacts. For high-low differences, positive numbers indicate greater delta-alpha modulation during the fact epoch (relative to the baseline) for high performers. Statistical significance of differences between high and low performers was evaluated using a cluster-based (neighboring channels) random permutation approach (see section 2.4.5 [Calculation of Statistical Significance] for details of significance testing). P- and d-values denote statistical significance and effect size (Cohen’s d), respectively.

Figure S1

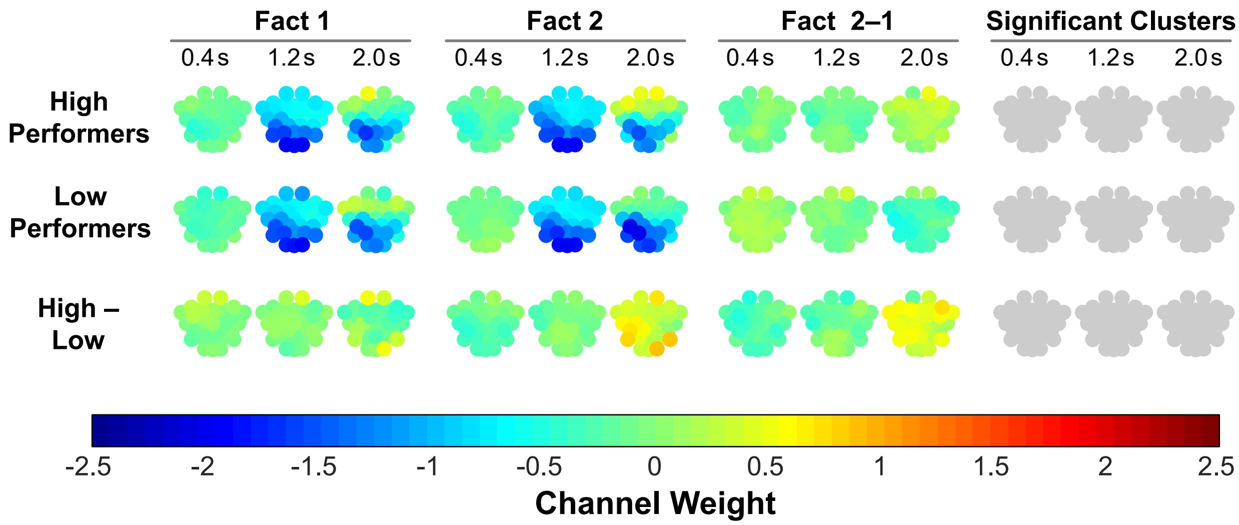
Fact 1
Fact 2
Fact 2–1
Significant Clusters
0.4 s
1.2 s
2.0 s
High Performers
Low Performers
High – Low
-2.5
-2
-1.5
-1
-0.5
0
0.5
1
1.5
2
2.5
Channel Weight

Figure S2

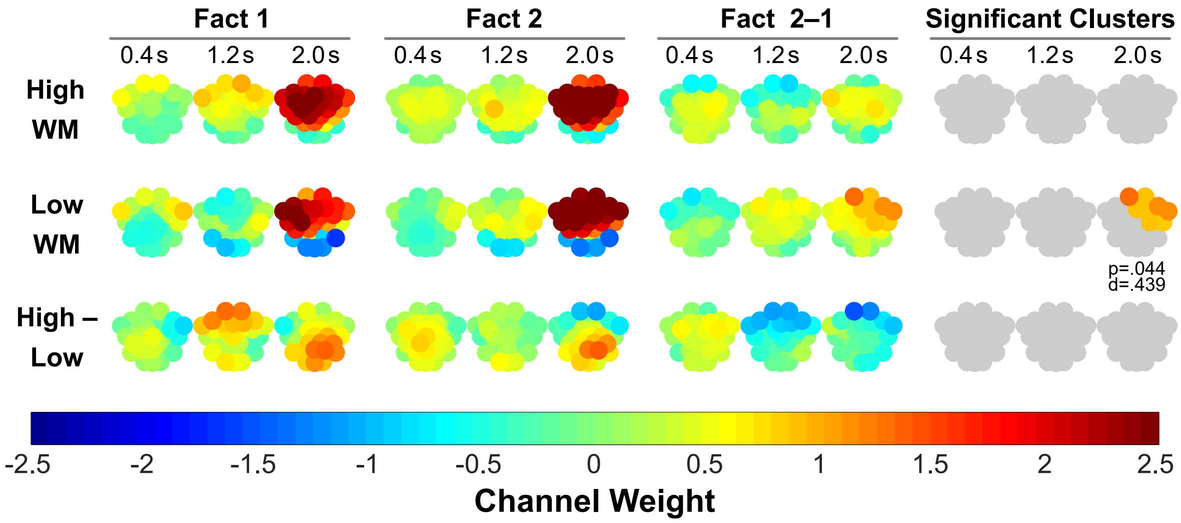
Fact 1
Fact 2
Fact 2–1
Significant Clusters
0.4 s
1.2 s
2.0 s
High WM
Low WM
High – Low
p=.044
d=.439
-2.5
-2
-1.5
-1
-0.5
0
0.5
1
1.5
2
2.5
Channel Weight

Figure S3

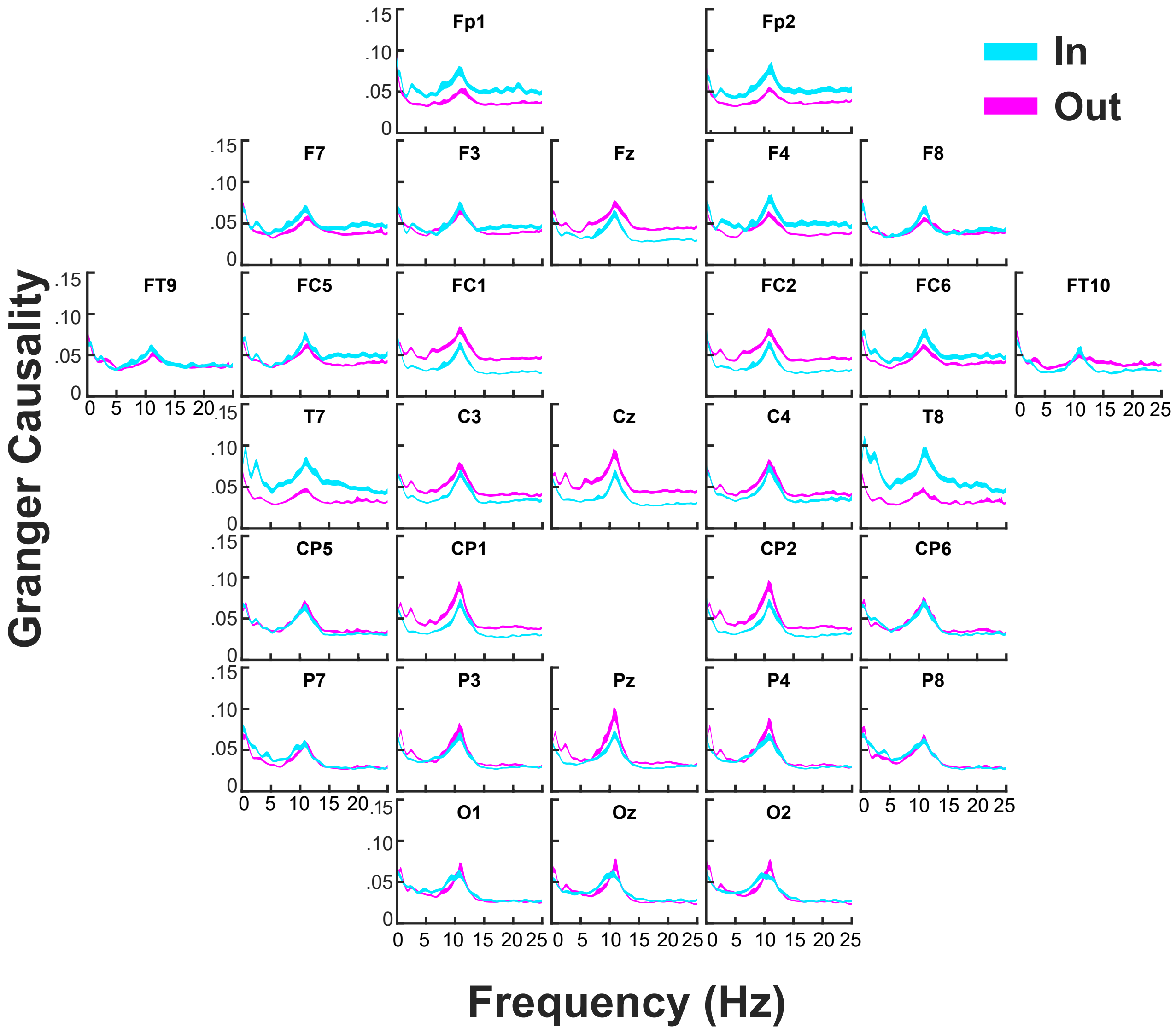

In
Out
Granger Causality
Frequency (Hz)
Fp1
Fp2
F7
F3
Fz
F4
F8
FT9
FC5
FC1
FC2
FC6
FT10
T7
C3
Cz
C4
T8
CP5
CP1
CP2
CP6
P7
P3
Pz
P4
P8
O1
Oz
O2
.15
.10
.05
0
0 5 10 15 20 25

Figure S4

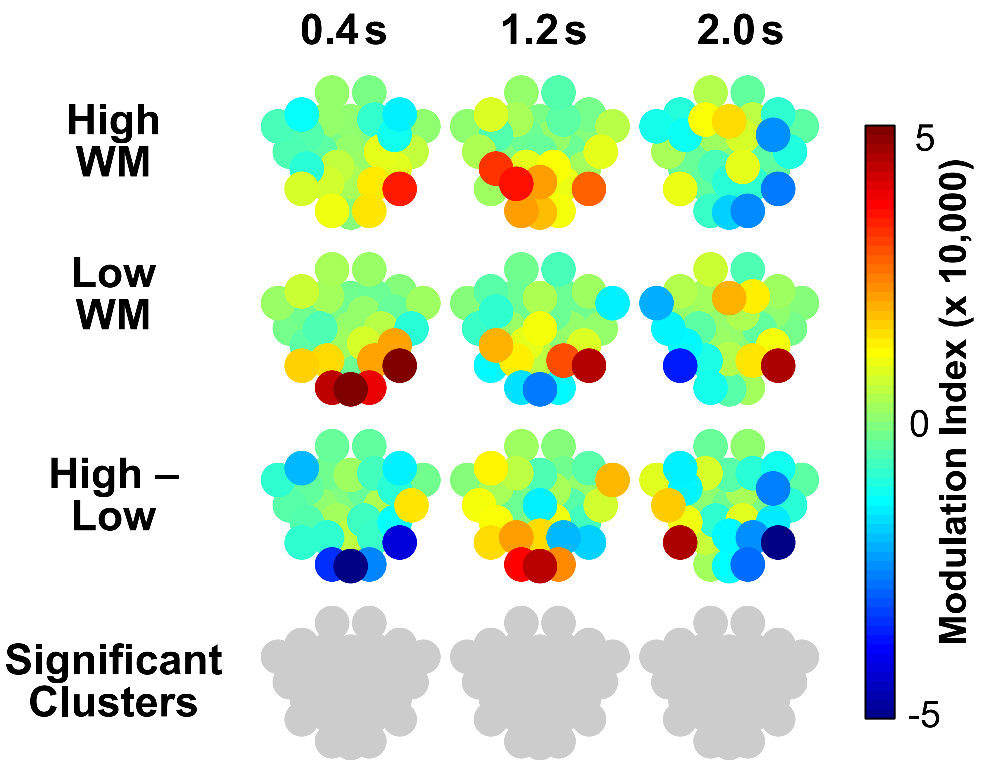
0.4 s
1.2 s
2.0 s
High WM
Low WM
High – Low
Significant Clusters
5
0
-5
Modulation Index (x 10,000)

Figure S5

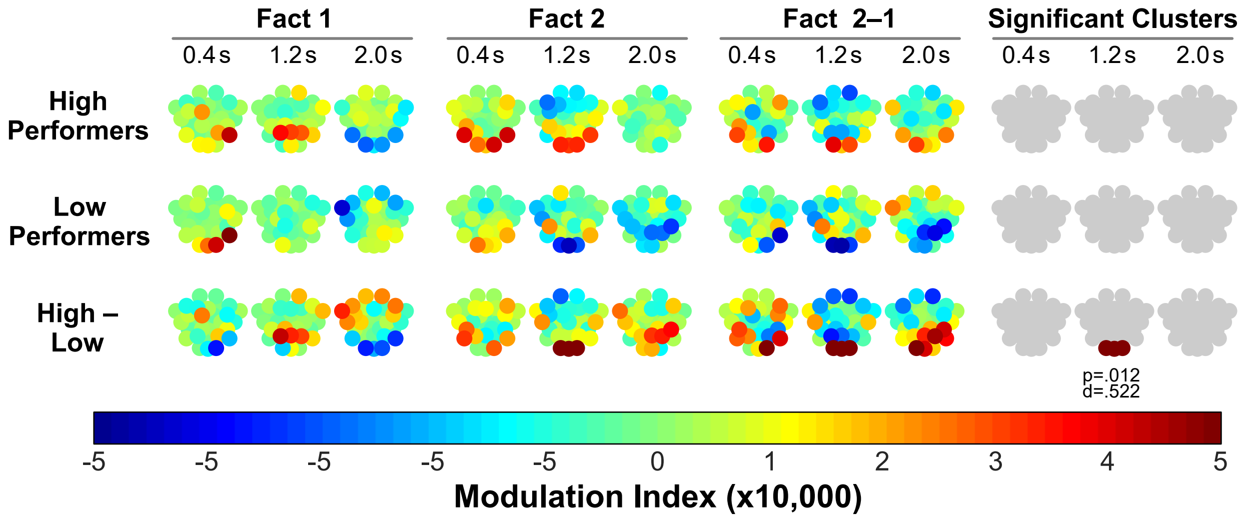
Fact 1
Fact 2
Fact 2–1
Significant Clusters
0.4 s
1.2 s
2.0 s
High Performers
Low Performers
High – Low
p=.012
d=.522
-5
-5
-5
-5
-5
0
1
2
3
4
5
Modulation Index (x10,000)
